# The Medicine for a Changing Planet curriculum: a mixed methods evaluation

**DOI:** 10.3389/fpubh.2026.1737962

**Published:** 2026-02-04

**Authors:** Noelle A. Benzekri, Alice H. Tin, Seth D. Judson, Kelsey Ripp, Michael Xie, Erika Veidis, Michele Barry, Peter Rabinowitz

**Affiliations:** 1 Division of Allergy and Infectious Diseases, Department of Medicine, University of Washington, Seattle, WA, United States; 2 Department of Health Systems and Population Health, University of Washington, Seattle, WA, United States; 3 Swedish Cherry Hill Family Medicine Residency Program, Seattle, WA, United States; 4 Division of Infectious Diseases, Department of Medicine, David Geffen School of Medicine at UCLA, Los Angeles, CA, United States; 5 Center for One Health, University of Global Health Equity, Butaro, Rwanda; 6 Baylor College of Medicine, Houston, TX, United States; 7 Center for Innovation in Global Health, Stanford School of Medicine, Stanford, CA, United States; 8 Center for One Health Research, University of Washington, Seattle, WA, United States; 9 Department of Environmental and Occupational Health Sciences, Department of Global Health, University of Washington, Seattle, WA, United States

**Keywords:** climate change, competencies, curriculum, environment, medical education, mixed methods, One Health, Planetary Health

## Abstract

**Background:**

There is a need to more fully integrate Planetary Health and One Health principles into current medical education programs. The Medicine for a Changing Planet (MCP) curriculum is a set of online, freely available, competency-based clinical cases developed by the authors to address this gap. This study was conducted to pilot and evaluate the MCP curriculum using a mixed methods approach.

**Materials and methods:**

Residents were invited to participate in interactive didactic sessions during which the authors piloted the content of the 11 MCP cases and collected feedback. Each session included a pre-test, case delivery using one of the MCP slide sets, and a post-test. Feedback was obtained using a structured questionnaire and focus group discussions.

**Results:**

Total attendance across all didactic sessions was 87, including 57 unique participants. The median pre-test score was 67%, and the median post-test score was 83% (*p* < 0.01). All participants agreed that understanding how environmental changes, including climate change, impact human health is important. The majority agreed that the case enhanced their understanding of the topic and that the material was useful for their clinical practice. All agreed that the case material and clinical concepts were clear. Themes that emerged from focus group discussions included (1) the importance of integrating Planetary Health and One Health principles into medical education, (2) enhancing relevance to the local context, (3) balancing detail with overarching principles, (4) maximizing the impact of the “Beyond the Clinic” and “Call to Action” sections, (5) optimizing content delivery, and (6) balancing action and despair in the face of a changing environment.

**Conclusion:**

The MCP curriculum was effective in increasing understanding and recognition of how Planetary Health and One Health approaches can improve patient care, and there was strong demand to integrate this content into medical education.

## Introduction

Human health depends on the health of our environment. As a result of climate change, pollution, deforestation, and multiple other environmental challenges, we are faced with shifting patterns of disease and new threats to human health, wellbeing, and security (1). In this era of rapid change, the role of physicians and other health professionals is evolving. The future of medicine requires physicians capable of systems thinking, urgent action, and interdisciplinary approaches to healthcare and ecological stewardship (2–21).

The fields of Planetary Health and One Health highlight the interdependence of human, animal, plant, and environmental health and emphasize that comprehensive patient care requires attention to ecological systems (4, 22–25). One Health is defined as “an integrated, unifying approach that aims to sustainably balance and optimize the health of humans, animals, plants, and ecosystems.” It recognizes that “the health of humans, domestic and wild animals, plants and the wider environment (including ecosystems) are closely linked and interdependent” (22). Planetary Health is a more recent “solutions-oriented, transdisciplinary field and social movement focused on analyzing and addressing the impacts of human disruptions to Earth's natural systems on human health and all life on Earth” (23). Despite growing recognition of the importance of these principles for contemporary clinical practice, Planetary Health and One Health concepts are not emphasized in medical school curricula or Graduate Medical Education programs (26–31).

The Medicine for a Changing Planet (MCP) curriculum (https://www.medicineforachangingplanet.org) is a set of 11 online, freely available, competency-based clinical cases that we developed to address this critical gap in current medical education (Table 1) (32, 33). Each case focuses on a distinct high-priority clinical topic, spanning across a range of medical disciplines, including water-related disasters, air pollution, vector-borne diseases, toxic exposures, emerging zoonoses, ecoanxiety, pets and other animal sentinels, pandemic preparedness, food security, displacement and refugee health, and heat-related illness Continuing Medical Education (CME) credits can be earned for each case.

**Table 1:** The Medicine for a Changing Planet cases.

**Case title**	**Clinical competencies**	**Clinical stem**
Toxic exposures	1. Take a medical history that screens for toxic exposures 2. Develop a differential diagnosis and diagnostic testing for suspected heavy metal toxicity 3. Recognize sentinel cases (human and animal) for toxic exposure 4. Apply a host-environment approach to the management of a toxic exposure	In a rural area of West Africa, a 3-year-old child is admitted to the local hospital with abdominal pain and decreased responsiveness after a witnessed seizure
Pandemic preparedness	1. Know how to perform a comprehensive history, exam, and diagnostic testing for fever in a returning traveler 2. Understand how to diagnose emerging zoonoses and high-consequence pathogens 3. Apply measures to ensure healthcare and laboratory worker safety 4. Communicate and coordinate with public health outbreak response	A 44-year-old woman presents to your primary care clinic with severe headache, fever, chills, nausea, vomiting, diarrhea, and a diffuse rash. She just traveled back from Uganda, where she went on a safari
Air pollution	1. Perform a detailed (and locally adapted) environmental history to identify environmental hazards that relate to the patient's presentation 2. Recognize sentinel cases of potential air pollution-related health effects. 3. Demonstrate the use of an environmental hazard risk assessment framework to guide clinical decision-making 4. Demonstrate how to obtain, interpret, and utilize data on air quality to impact patient care 5. Create preventive treatment plans that include improving host vulnerability (host factors) and reducing environmental exposures to air pollution.	A 55-year-old woman in rural Rwanda presents to the hospital with progressively worsening dyspnea and lower extremity and abdominal swelling. She cooks daily on an indoor stove fueled by harvested wood
Vector-borne diseases	1. Integrate the history of vector exposure into the patient's history 2. Develop a differential diagnosis for vector-borne disease 3. Understand and prioritize diagnostic testing for vector-borne disease 4. Understand populations vulnerable to vector-borne disease 5. Access and integrate risk maps, surveillance data, and local outbreak and entomology data to improve diagnostic accuracy 6. Detect sentinel cases of vector-borne disease 7. Integrate weather data, extreme events, and local environmental factors to inform the differential diagnosis 8. Access resources to guide clinical management of climate-sensitive infections 9. Educate patients, communities, and policymakers about prevention and the links between vector-borne disease and climate change	A 30-year-old female presents to an emergency room in Miami, Florida, with fever, headache, and flank pain
Pets and other animal sentinels	1. Understand what to do when patients give a history of sick animals in the house or nearby 2. Develop communication strategies with veterinarians and animal health experts 3. Recognize sentinel cases (human and animal) 4. Understand how to notify regarding sentinel cases as a first step to improving shared environments	A 60-year-old woman is admitted to the hospital in the Northeastern US with fever, headache, nausea, vomiting, fatigue, and altered mental status. She reports that her dog died the week before, which also had a fever and neurological problems
Emerging zoonoses	1. Integrate the history of animal contacts and environmental exposures in the development of a differential diagnosis that includes zoonoses 2. Select appropriate diagnostic testing for emerging zoonotic diseases 3. Understand the origins of zoonotic outbreaks, including modes of transmission and sources of spillover 4. Identify community health interventions that can limit zoonotic disease spread	A 28-year-old man presents to the ER in Kerala, India, with confusion, fever, and vomiting. His relatives, some of whom catch wild animals and collect fruit, are also in the ER and have similar symptoms
Ecoanxiety	1. Assess potential climate-related and environmental-driven emotional factors 2. Differentiate between eco-anxiety, work burnout, and other confounding medical conditions 3. Differentiate the normal range of emotional responses to our changing environment from clinically significant presentations 4. Develop the capacity to teach patients behavioral strategies for the management of eco-anxiety 5. Develop the capacity to counsel patients on managing exposure to troubling news and environments 6. Identify resources for referrals when indicated	You are seeing a 28-year-old patient in the Western U.S. who vaguely reports “not feeling well” and is generally concerned about her health. As you take a history, you find that she has significant concerns about climate change and the environment
Water-related disasters	1. Take a medical history that screens for water-related health risks 2. Integrate screening for water-related health risks into the physical exam, including those requiring surgical intervention 3. Detect and manage sentinel cases of water-related disease 4. Recognize and manage necrotizing soft tissue infections (NSTIs) as a surgical emergency	A 27-year-old male presents with severe pain in his right leg 7 days after a typhoon resulted in severe flooding in his community in the Philippines
Food security	1. Combine information obtained using validated tools with knowledge of local diets and food sources to identify patients with food insecurity 2. Assess environmental and structural barriers to food access and dietary diversity 3. Apply clinical evaluation skills to detect signs and symptoms of malnutrition 4. Propose evidence-based and culturally appropriate dietary interventions that take into account both health and environmental considerations	A 50-year-old male living in a small town in rural Alaska presents for diabetes follow-up. This patient has a history of hypertension and diet-controlled diabetes
Displacement and Refugee Health	1. Identify the leading health conditions among resettled refugees and migrant populations 2. Access and apply the CDC guidelines for immigrant and refugee health in the clinical management of a newly arrived refugee 3. Screen for, diagnose, and treat specific health conditions of concern among refugees presenting for care 4. Provide preventive care for resettled persons 5. Provide culturally competent care 6. Integrate up-to-date resources and guidelines into the clinical management of refugees and migrant patients	A 28-year-old woman recently arrived in the US from Somalia presents to your primary care clinic to establish care
Heat-related illness	1. Analyze current epidemiology regarding heat-related morbidity and mortality, and how this is expected to change with climate change 2. Articulate how heat exposure impacts existing health inequities 3. Connect the impact of increasing global temperatures to ecosystem health, and how this, in turn, impacts human health 4. Recognize the clinician's role in climate change mitigation and adaptation to prevent worsening impacts of heat on human health	You are a general practitioner in London. The heat index today is 107°F (41.6 °C). You are seeing an 83-year-old woman with congestive heart failure, atrial fibrillation, and chronic kidney disease who is not feeling well

While environmental changes impact every region of the globe, climate change is expected to cause disproportionate harm in low-income countries and among low-income populations in high-income countries (34–37). As such, we intentionally sought to include cases from both high- and low-resource settings, developed with input from healthcare providers worldwide.

Clinical competencies were established for each case, emphasizing higher-level cognitive domains according to revised Bloom's Taxonomy (38). The cases were developed using a standardized template and consist of (1) a clinical vignette followed by an interactive clinical case presentation, (2) a “Beyond the Clinic” section that emphasizes the importance of collaboration with public health agencies and provides context for how environmental changes are impacting communities and regions, (3) a “Call to Action” section that suggests steps that clinicians can take to respond to planetary health challenges, (4) a knowledge assessment consisting of 4–6 questions, and (5) additional references and resources. Each case also introduces and highlights the importance of the “Social-E” history, which expands upon the traditional social history to include factors related to the patient's environment (39).

This study was conducted to pilot and evaluate the Medicine for a Changing Planet curriculum using a mixed methods approach.

## Materials and methods

This study was conducted using a convergent mixed methods design. Trainees in the Family Medicine Residency Program at Swedish Cherry Hill in Seattle, Washington, were invited to participate in interactive didactic sessions during which we piloted the content of the 11 Medicine for a Changing Planet cases and elicited feedback. Sessions were held from August 2023 to March 2025.

We piloted one case per session. Trainees were informed of the date, time, and location of each session using a program-wide email announcement. Each session consisted of a pre-test, case delivery using one of the Medicine for a Changing Planet slide sets, and a post-test. Feedback was obtained immediately after the case session using a structured questionnaire and a focus group discussion. Case sessions and focus group discussions were facilitated by the first author, who is a medical educator and infectious diseases specialist with expertise in qualitative methods.

The pre-test, post-test, and structured questionnaire were administered using paper data collection forms. The pre-test and post-test consisted of 5–6 knowledge assessment questions. The structured feedback questionnaire was developed iteratively, with questions added based on feedback from the preceding case sessions. This process continued through case 3, after which no further modifications were made to the questionnaire. The questionnaire consisted of 6–13 questions, including open-ended questions and Likert-scale items (scored as 1= strongly disagree, 2= disagree, 3= neither agree nor disagree, 4= agree, and 5= strongly agree). Quantitative data were analyzed using SPSS Statistics 29. Descriptive analysis was performed for all variables. The Wilcoxon signed-rank test was used to compare pre-test and post-test scores. Missing data were excluded from the analysis. *P*-values of < 0.05 were considered significant. Focus group discussions were facilitated using a topic guide. Detailed notes were taken throughout the discussions. Discussions were audio-recorded, transcribed, and uploaded into Atlas.ti (v23.4). Qualitative data were analyzed using thematic analysis and inductive coding (40).

This study was reviewed by the University of Washington and the Swedish Cherry Hill IRB committees and was deemed exempt (Category 1).

## Results

The total attendance across all sessions was 87 trainees, including 57 unique participants. The overall response rate among all program residents was approximately 88%. The mean number of participants per case was 8 (range 3–18). The mean duration of case delivery was 38 minutes (range 29–46). The mean duration of the focus group discussion was 25 minutes (range 11–47).

### Knowledge assessment

The median pre-test score across all cases was 66.7%, and the median post-test score across all cases was 83.3% (*p* < 0.001) (Table 2).

**Table 2:** Knowledge Assessment, median test score.

**Case**	**Pre-test**	**Post-test**	*p*-value^*^
All cases	66.7	83.3	< 0.001
Toxic exposures	66.7	91.7	
Pandemic preparedness	50.0	66.7	
Air pollution	83.3	75.0	
Vector-borne diseases	66.7	83.3	
Pets and other animal sentinels	83.3	100	
Emerging zoonoses	50.0	83.3	
Ecoanxiety	58.3	100	
Water-related disasters	50.0	50.0	
Food security	50.0	83.3	
Displacement and Refugee Health	50.0	100	
Heat-related illness	–	83.3	

^*^Related-samples Wilcoxon signed rank test; N = 69.

### Structured feedback

For data obtained using the structured questionnaire, the words “this topic” were replaced by the specific case topic when the questionnaire was administered (Table 3).

**Table 3:** Perceptions^*^.

**1%−24% of respondents** **25%−49% of respondents** ** 50%−74% of respondents** ** 75%−100% of respondents**	**Understanding how environmental changes, including climate change, impact human health is important **	**I would like our curriculum to include more information about the impacts of this topic^a^ on human health^b^**	**The case increased my understanding of how this topic^a^ can impact human health **	**Understanding how this topic^a^ impacts human health is important^b^**	**The case increased my understanding of the approach to this topic^a^**	**The case material is useful for my current clinical practice **	**The case material could be useful for my future clinical practice **	**The case material and clinical concepts were clear^c^**	**The “Patient Case” section was useful and contributed to my learning^d^**	**The “Beyond the Clinic” section was useful and contributed to my learning^d^**	**The “Call to Action” section was useful and contributed to my learning^d^**
**All cases**, ***n*** = **87**											
Disagree					2%	5%					
Neither agree nor disagree		7%	7%		2%	12%	1%		3%	8%	14%
Agree	29%	37%	52%	46%	48%	54%	53%	57%	52%	60%	49%
Strongly agree	71%	56%	41%	54%	48%	30%	46%	43%	45%	32%	37%
**Toxic exposures**, ***n*** = **18**											
Disagree						11%					
Neither agree nor disagree		6%	11%		6%	11%				11%	17%
Agree	28%	33%	56%	28%	50%	72%	78%	61%	44%	56%	50%
Strongly agree	72%	61%	33%	72%	44%	6%	22%	39%	56%	33%	33%
**Pandemic preparedness**, ***n*** = **14**											
Disagree						7%					
Neither agree nor disagree		14%	14%			14%				7%	21%
Agree	43%	43%	57%	57%	57%	71%	71%	71%	71%	57%	50%
Strongly agree	57%	43%	29%	43%	43%	7%	29%	29%	29%	36%	29%
**Air pollution**, ***n*** = **4**											
Neither agree nor disagree						25%	25%		25%		25%
Agree	75%	50%	100%	75%	100%	50%	50%	100%	50%	75%	50%
Strongly agree	25%	50%		25%		25%	25%		25%	25%	25%
**Vector-borne diseases**, ***n*** = **3**											
Agree			67%		67%	33%		33%		33%	33%
Strongly agree	100%	100%	33%	100%	33%	67%	100%	67%	100%	67%	67%
**Pets as sentinels**, ***n*** = **17**											
Disagree					6%	6%					
Neither agree nor disagree		12%			6%	24%			6%	12%	6%
Agree	47%	47%	65%	59%	53%	41%	59%	59%	59%	65%	59%
Strongly agree	53%	41%	35%	41%	45%	29%	41%	41%	35%	23%	35%
**Emerging zoonoses**, ***n*** = **3**											
Neither agree nor disagree						33%					
Agree	33%				67%	33%	67%	67%	67%	67%	67%
Strongly agree	67%	100%	100%	100%	33%	33%	33%	33%	33%	33%	33%
**Ecoanxiety**, ***n*** = **4**											
Neither agree nor disagree											25%
Agree		50%	25%	50%	25%	75%	25%	25%	50%	75%	25%
Strongly agree	100%	50%	75%	50%	75%	25%	75%	75%	50%	25%	50%
**Water-related disasters**, ***n*** = **3**											
Agree						50%				50%	
Strongly agree	100%	100%	100%	100%	100%	50%	100%	100%	100%	50%	100%
**Food security**, ***n*** = **3**											
Agree		33%	33%		33%	67%	33%	33%			
Strongly agree	100%	67%	67%	100%	67%	33%	67%	67%			
**Refugee health**, ***n*** = **5**											
Agree					25%	25%	25%	25%			
Strongly agree	100%		100%		75%	75%	75%	75%			
**Heat-related illness**, ***n*** = **13**											
Neither agree nor disagree			8%								
Agree	8%		42%		33%	33%	25%				
Strongly agree	92%		50%		67%	67%	75%				

^*^The statement, “The amount of time spent on the case was appropriate,” was also included in the structured questionnaire. Responses are described in the results section of the text.

^a^The words “this topic” were replaced by the specific case topic when the questionnaire was administered.

^b^This statement was not yet included at the time of the “heat-related illness” or “refugee health” case sessions.

^c^This statement was not yet included at the time of the “heat-related illness” case session.

^d^This statement was not yet included at the time of the “heat-related illness,” “refugee health,” or “food security” case sessions.

“Understanding how environmental changes, including climate change, impact human health is important”: All participants either agreed (29%) or strongly agreed (71%).

“I would like our curriculum to include more information about the impacts of *this topic* on human health”: The majority of participants either agreed or strongly agreed. No participants disagreed.

“The case increased my understanding of how *this topic* can impact human health”: The majority of participants either agreed or strongly agreed. No participants disagreed.

“Understanding how *this topic* impacts human health is important”: All participants either agreed or strongly agreed.

“The case increased my understanding of the approach to *this topic*”: The majority of participants either agreed or strongly agreed. For the “pets as sentinels” case, 6% disagreed.

“The case material is useful for my current clinical practice”: The majority of participants either agreed or strongly agreed. For the “air pollution” case, 25% neither agreed nor disagreed, for the “pets as sentinels” case, 24% neither agreed nor disagreed, and for the “emerging zoonoses” case, 33% neither agreed nor disagreed. In the “toxic exposures” case, 11% disagreed; in the “pets as sentinels” case, 6% disagreed; and in the “fever in a returning traveler” case, 7% disagreed.

“The case material could be useful for my future clinical practice”: The majority of participants either agreed or strongly agreed. For the “air pollution” case, 25% answered neither agreed nor disagreed. No participants disagreed.

“The case material and clinical concepts were clear”: All participants either agreed or strongly agreed.

“The Patient Case section was useful and contributed to my learning”: The majority of participants either agreed or strongly agreed. For the “air pollution” case, 25% neither agreed nor disagreed. No participants disagreed.

“The Beyond the Clinic section was useful and contributed to my learning”: The majority of participants either agreed or strongly agreed. No participants disagreed.

“The Call to Action section was useful and contributed to my learning”: The majority of participants either agreed or strongly agreed. For the “air pollution” and “ecoanxiety” cases, 25% neither agreed nor disagreed. No participants disagreed.

### Time allocation

In the majority of cases, participants agreed that the time spent on the case was appropriate. For the “vector-borne diseases” case, 67% would have preferred more time, and for the “water-related disasters” case, 50% would have preferred more time.

### Themes from focus group discussions

1. **Importance of integrating Planetary Health and One Health principles into medical education:** Current medical education was perceived as inadequate in the face of current environmental challenges. The integration of a Planetary Health and One Health curriculum into medical education was considered both important and timely.

- “*Medical school really didn't prepare me to tackle some of the impacts that I foresee of the effect of climate change on global health as a whole or just global health in my community.” (Case 4)*
- “*It makes sense that there are changes that happen in our environment that affect downstream health outcomes for people and animals. The details of how all of those changes impact our health are important to understand… but it's hard to know, in the scope of our training, what do we need to be looking out for more broadly in our patient population? What things have we been taught over the long haul, over all these years of clinical training, that are no longer true because of the changing environment? Because of the changing environment, what things do we need to start broadening our thinking about?” (Case 10)*
- “*As far as the curriculum as a whole, I think it should be a requirement to some degree for preparing future physicians.” (Case 4)*
- “*Because it's a thing that is actually happening and, as is the point in this case, [the diagnosis] was something that we didn't think was on the differential because the story didn't quite fit. And so you might erroneously rule something out, whereas the environment and climate are changing in ways that are actually impacting human health and forcing us to [revise] our differential.” (Case 7)*
- “*Even if you don't believe in climate change, there's a conversation about climate change. So having this in the curriculum makes sense regardless of your position.” (Case 6)*

2. **Enhancing relevance to local context:** A recurring recommendation was that the curriculum would be improved by providing greater emphasis on local context. While participants appreciated the global perspective, they noted that some cases would be more valuable if they included clinical scenarios more applicable to what they might see in their own practice settings. In addition, providing information on reliable resources that can be used to stay informed about the local context was suggested.

- “*I was thinking I would prefer a case that's more applicable to where we are and the clinics that we serve… I understand that this is a global sort of project, but I think just to help us keep an eye out for things that we are likely to see ourselves here. I think having more of this closer to home, to our areas, would benefit us.” (Case 8)*
- “*I really appreciate the context of this particular example, [but] I wonder as this rolls out, if they could be developed such that it is a case of someone that [we] would see at the clinic. So while it's big picture teaching about climate change and food security, it also gives tangible resources at the end that are specialized to that particular [location].” (Case 3)*
- “*Even if, like in the slide deck that everyone got, even if it doesn't have the things that are specifically local, including just a prompt to think about it, I feel like that would be helpful.” (Case 5)*
- “*I hope to find generalizable titbits or things that I can do, ways to be thinking about things, so that I can improve my understanding of patient care. So, [for example] how can I tell what exposures are common in my environment? What is the best way to know what diseases are increasing in prevalence in my community? What databases should I be looking at or reading to keep on top of the zoonotic illness burden where I live? Because there's going to be some places we go where this specific case is not going to be as relevant, but there are always skills within this that are relevant.” (Case 10)*
- “*It underscored the importance of coming into a new area and understanding the social factors, the environmental factors, and being mindful of all the aspects of every region that you serve… So I think this was a good case for that because it kind of underscored the importance of understanding the specific factors of a community in different geographical regions.” (Case 5)*

3. **Forest for the Trees—Balancing detail with overarching principles:** There were mixed perspectives about the optimal balance of detail to include in each case vs. emphasizing overarching principles.

- “*I think the piece that I was kind of struck by during the case was this question of, what is the point of these data gathering slides, when really, I would have been like, “Hello infectious disease colleagues, I am out of my depth.” (Case 9)*
- “*There was a specific point when we got into the details of all the testing available and what's the most sensitive and specific. I didn't follow it, to be perfectly honest… I think I'm a little lost in the weeds here. And so I guess my question is, at what point is this something that I should be vaguely aware of, super aware of? Do I just call the person who's working in the lab and say, what do we have access to? I guess my question is about the Forest for the Trees here, like, what is the most helpful level of detail for someone who is not an infectious disease doctor?” (Case 9)*
- “*One thing I was curious for is more education around a forest level view of the environmental pressures that are causing mosquitoes to spread, or just some more forest level view concepts, because I think the clinical teaching points are helpful, and at the same time, I think for the section of, like, call to action, I feel like I would have a better understanding of what I'm being called to act if I just had a better understanding of what's driving some of these changes.” (Case 7)*

4. **Maximizing the impact of the “Beyond the Clinic” and “Call to Action” sections:** There were mixed perceptions regarding the utility of the “Beyond the Clinic” and “Call to Action” sections. The emphasis on interprofessional communication and partnership in the “Beyond the Clinic” was considered increasingly important. For example, the concept of animals as sentinels was viewed as novel and highly relevant. However, providing more information regarding concrete mechanisms to facilitate communication with other health professionals, including public health experts and veterinarians, was suggested.

- “*The idea of animals being sentinel cases, I think it's an important reminder as well, that we're not the only [providers] that are there taking care of animals or taking care of [patients], and there are animals involved. That was an important reminder as well. (Case 7)*
- “*I got the overall message, but still I have the question of, wait, if that were me, as the primary clinician, after seeing this, would I know what to do? And right now, I don't think I know how to contact [these agencies] or if I am [even] the one to do it... You know, the practical, the more “how to do it”. (Case 11)*
- “*I would love seeing a little bit more information on if you are somewhere with no resources, do I call the CDC? Do I call the public health person? Or, where online do I go to figure out the information when there is no treatment? Or how do you figure out, because you're not going to probably give information to every small community that you're going to ever present this to, but helping them know how they figure that out for themselves, perhaps just [including] the county public health office, the name of who you're looking for, and phone number.” (Case 11)*

While the “Call to Action” concept was viewed as important, participants felt that it would be more effective if it contained more local resources and suggestions for local action.

- “*This is a political issue. And so to make it apolitical doesn't make sense.” (Case 8)*
- “*I was just thinking… about making things maybe more important to the here and now and for us, so utilizing the case and taking it a step further, to show what sort of actions are being done or what you can do within the community, and things that are ongoing either in legislature or within the community itself that relate back to the case, [to] just further bring home the message and the call to action and have it be a little bit more important.” (Case 8)*
- “*I do think that this is one of those places where action feels so big and insurmountable… But the more concrete and specific you are, the more you can take a step into this. I think taking the first step is usually the hardest part for people to feel like they can get involved. So making some small, concrete, tangible options along with the bigger, “we got to work on climate change and displacement and deforestation and flooding [etc]...” (Case 9)*
- “*I think the call to action piece is probably the most generalizable… I think the idea that I could develop a protocol or figure out a way to talk to veterinarians is a new concept and something that I think could be explored more, whereas I think that we covered the patient case in enough detail.” (Case 10)*

Importantly, consideration for the stage of training during which the curriculum is delivered was viewed as critical for maximizing the value of the “Call to Action” section.

- “*That's why I'm like, ‘Give me something small I can do while I'm still tending to my basic needs.' And then when I'm an Attending, and I have more time, I'll do bigger things.” (Case 9)*
- “*Depending on the audience, there's probably a spectrum of people who engage with this material who are really inclined toward the ‘Call to Action' section, vs. others who are like, ‘Oh, this is another thing I have to do' and if someone's in this phase of burnout, that could just contribute to another overwhelming sense that, okay, I have another thing to do. So that could be a negative impact.” (Case 6)*

5. **Optimizing content delivery:** A longitudinal curriculum using a small group format was considered the optimal modality for integrating the curriculum into training programs.

- “*I think the dream is to have climate change curriculum woven throughout your training.” (Case 6)*
- “*It should be something that's [delivered] over time, integrated into their thought process or, you're given opportunities to apply it across multiple contexts, preclinical, clinical, and finally when you're actually practicing.” (Case 8)*
- “*I think it's reasonable to be part of a required training and I think that there are pretty seamless ways that it can be built into other themes and topics, and lectures. So, I think it could be its own curriculum, but I think that a longitudinal [delivery], building it in repetition over time as it relates to the different topics we already go through, could be good.” (Case 5)*

The use of discussion prompts throughout the cases was considered highly valuable and well-suited to this type of content.

- “*I do like the discussion format. I like to hear how other people are thinking and hearing their clinical reasoning. And I really liked having [the instructor's] input as well, and I think that just makes me a better doctor. And I'm a tired resident, so I think the more things are built into a structured environment, the more I appreciate it. But I also really do like just how easy it is to access this training and information on a website that I can revisit later, or I can do a self-study. So I like having the option of both. But I do think that the discussion aspect is very valuable.” (Case 7)*
- “*I picture didactics with residents and faculty in a discussion format. I think part of it is because these prompts would be so provoking and thoughtful in an environment like that. I think that's where I would see a lot of benefit that I feel like would be missed out on if it were just me clicking through them on a computer, solitarily. Not to say that it wouldn't be a benefit to do so as well, if that was the opportunity presented.” (Case 5)*

Participants noted that trainees in their R2 or R3 years of training, rather than during intern year, may be more receptive to the content.

- “*I think part of that too is now that I'm an R3 and significantly less burnt out compared to my first year. I can actually allow myself to contemplate such an existential dread that is climate change.” (Case 3)*

There was a preference for more tables, diagrams, images, and frameworks to more effectively conceptualize and apply complex principles. There was appreciation for slides that synthesized the material and could be printed or downloaded for use as a high-yield reference in the clinic. Developing more of these types of “reference slides” was suggested.

- “*More visual slides… I think the slides have a lot of helpful information, but I think fewer words would allow me to take those few keywords with me, vs. a large body of text. And then the visuals just seem to stick in my mind better.” (Case 7)*
- “*I really appreciated the visual aids and the way that the graphs were made to help us think… because that's a page on the slide that I can just print out. Like, here are measures that work. It's visual.” (Case 1)*
- “*I found the slide on the questions to ask, how to think of a framework on exposure to animals, and all that, really helpful.” (Case 9)*
- “*I think one thing that would be even more helpful as a tool to translate that to clinical practice would be if we could organize things in a way that we could take a picture of, or that generates a handout, or a reference… If there was some kind of graphic that could be taken from it, it would be even more helpful.” (Case 11)*
- “*The graphs were helpful just to see the sort of increase in mosquito-borne illnesses. That was really helpful to see. It was very stark. I think there were a lot of tables there which kind of compared different symptoms across different Arboviruses, and I think that was helpful.” (Case 7)*
- “*[I would have liked to have seen] a picture of the rash, and some pictures of the other defining physical exam features of the other illnesses. Even if it's not a picture of this patient.” (Case 11)*

6. **Balancing action and despair in the face of a changing environment:** Participants expressed a feeling of despair in the face of climate change and other environmental challenges. The ability to confront these challenges through clinical care and concrete actions helped to alleviate feelings of cynicism and hopelessness.

- “*I think it also feels really overwhelming. I really appreciate that, even though this is a very broad topic, trying to have some narrowness, because feeling very hopeless is a common side effect of having these conversations. I think that's also why we choose not to recognize it in the clinic. It's not just the time-limitedness of our visits. It's also that it's devastating to actually think about the reality every single time, and you can't do that and also keep your heart alive.” (Case 3)*
- “*At this current stage, feeling grateful to have this opportunity… but overall feeling a little pessimistic about the hopelessness of the topic in general. I think today did help me understand in more concrete terms how I can think about [this topic] in a less hopeless, devastating way.” (Case 3)*

## Discussion

The Medicine for a Changing Planet curriculum effectively increased understanding and recognition of how Planetary Health and One Health approaches can improve patient care, and there was strong demand to integrate this content into medical education. This study provided a critical opportunity to obtain actionable feedback on the curriculum's strengths and to suggest improvements and implementation strategies.

### Importance of the topic and integration into medical training

The overarching principle of understanding how environmental changes, including climate change, affect human health was considered important, as was understanding the impacts of each specific case topic. There was strong demand for these topics to be included in medical training. Trainees suggested that the optimal strategy for integrating the content into the residency training program would be to implement a longitudinal thematic thread. There was a preference for delivering the curriculum in a small-group or Problem-Based Learning (PBL) format rather than through lectures or self-directed learning, as the discussion enabled by the small-group format was considered highly valuable. An important concern was ensuring that the content was offered at a stage of training when residents are not predominantly consumed by inpatient clinical responsibilities and stressors, such as during the R2 and R3 years, rather than the intern year.

### Applicability to clinical practice

While the content was perceived as useful for both current and future clinical practice, the predominant suggestion for improvement across the curriculum was to place greater emphasis on local context in the cases. While the curriculum was initially developed to provide a range of clinical scenarios representing diverse communities across regions of the world, some participants felt this detracted from local relevance and made the cases less applicable to their clinical practice. As such, the cases would be optimized if educators intending to use the curriculum build upon the currently available slide sets. We suggest adding slides to include critical elements of the local context, such as environmental risk factors, epidemiology, and relevant case reports.

### Case structure and content

The cases were considered effective at clearly delivering the material and clinical concepts. Participants valued the interactive format and opportunities for discussion. As such, they suggested adding more prompts to the cases where these were included less frequently (vector-borne diseases, ecoanxiety, air pollution, and water-related disasters). The “Patient case” section was considered the most valuable. We did identify opportunities for improvement. There was a strong preference for more images and figures over text. Some cases were felt to be too detailed regarding aspects that seemed more relevant to specialists and consultants (emerging zoonoses and vector-borne diseases). Overall, the “Beyond the Clinic” section was considered useful; however, participants suggested more specific guidance on how to communicate and coordinate with local agencies and other professionals, such as veterinarians. Similarly, while the “Call to Action” section was perceived as useful, participants felt it could be improved by providing more specific, concrete steps and suggestions for local action. As above, the currently available slide sets can be modified to include contact information for community public health agencies and potential regional collaborators, as well as locally informed suggestions for community-level action.

### Limitations

This study was conducted among trainees in a family medicine residency program, who volunteered to participate in didactic sessions. As such, our findings may not be generalizable to trainees pursuing other specialties. Our study is further limited by sample size and the possibility that feedback obtained from focus group discussions may have been impacted by social desirability bias. Finally, participant enrollment was not random, and our findings are likely to be impacted by selection bias.

## Conclusion

There is an urgent need for graduate medical education programs to better prepare physicians to navigate the complex clinical consequences of a rapidly changing environment. The Medicine for a Changing Planet curriculum was highly acceptable and effective in increasing residents' understanding of how Planetary Health and One Health approaches can improve patient care. Integrating the Medicine for a Changing Planet curriculum into graduate medical education programs can help address a critical gap in current medical education.

## Data Availability

The datasets presented in this article may be made available by reasonable request to the corresponding author. Requests to access the datasets should be directed to: benzekri@uw.edu.
