# Supplementary material for: The Medicine for a Changing Planet curriculum: a mixed methods evaluation
Source: Front Public Health. 2026 Feb 4;14:1737962. doi: 10.3389/fpubh.2026.1737962 (PMC12913577; doi:10.3389/fpubh.2026.1737962)
Supplement: Supplementary file 1 [file Data_Sheet_1.pdf]

SUPPLEMENTAL CONTENT

I. KNOWLEDGE ASSESSMENTS

TOXIC EXPOSURES

1. Personality changes are associated with toxicity of:

   A. Lead
   B. Mercury
   C. Arsenic
   D. Iron

2. Toxic exposures associated with gold mining include:

   A. Mercury
   B. Lead
   C. Cobalt
   D. A and B

3. Chelation therapy for lead poisoning should be considered in children with blood levels above:

   A. 5 mcg/dL
   B. 25 mcg/dL
   C. 45 mg/dL
   D. 60 mg/dL

4. Most toxic exposures have clearly identifiable physical findings. TRUE or FALSE?

5. Animals may act as “sentinels” of human toxic exposure risk due to their:

   A. Greater exposure to the hazard compared to humans
   B. Greater susceptibility to the hazard compared to humans
   C. Lesser susceptibility to the hazard compared to humans
   D. A and B

6. When taking a patient history, what does the “E” stand for in “Social-E”?

PANDEMIC PREPAREDNESS

1. Which of the following infections have an incubation period of less than 21 days?

   A. Tuberculosis
   B. Hemorrhagic fever viruses
   C. Malaria
   D. Protozoal infections

2. Which of the following pathogens is least likely to cause petechiae?

   A. Marburg virus
   B. Neisseria meningitidis
   C. Dengue virus

D. Influenza virus

3. Which of the following viruses is a BSL-4 pathogen?

   A. Dengue virus
   B. MERS coronavirus
   C. Lassa virus
   D. West Nile virus
   E. Influenza virus

4. Which of the following infectious diseases have epidemic potential and require clinician reporting to public health authorities?

   A. Cholera
   B. Ebola virus disease
   C. Plague
   D. Brucellosis
   E. All of the above

5. You are evaluating a patient with a suspected viral hemorrhagic fever, which of the following tests has the least specificity?

   A. Serology via ELISA
   B. RT-PCR
   C. Virus isolation

6. When taking a patient history, what does the "E" stand for in "Social-E"?

<u>AIR POLLUTION</u>

1. The AQI accounts for particulate pollution as well as chemical contaminants such as volatile organic chemicals like formaldehyde. TRUE or FALSE?

2. Ways to build host resilience to air pollution include:
   A. Optimizing treatment of medical conditions
   B. Smoking cessation
   C. Vaccinations
   D. Peak flow measurements or other ongoing monitoring of respiratory status
   E. All of the above

3. Ways to manage patient environments to reduce exposure to air pollution include:
   A. Alternative cookstove technology that does not use biomass fuels.
   B. Promote change in transportation use in community
   C. Use of masks when appropriate
   D. A and C
   E. All of the above

4. AQI (air quality index) is reported in the same way in different countries. TRUE or FALSE?

5. The Social history (Social-E) can include questions about the effect of the environment on patient's respiratory status. TRUE or FALSE?

6. When taking a patient history, what does the "E" stand for in "Social-E"?

VECTOR-BORNE DISEASES

1. Aedes mosquitoes are the vector for which of the following diseases:

   A. Dengue
   B. Chikungunya
   C. Zika
   D. Yellow fever
   E. All of the above

2. Serology (IgM) is the most appropriate diagnostic for acute cases of vector-borne disease. TRUE or FALSE?

3. Characteristics of Aedes mosquitoes include all of the following except:

   A. Prefer urban areas
   B. Warming temperatures can increase abundance and spread
   C. Biting at night
   D. Invasive species

4. Sentinel cases of West Nile Virus have been reported in:

   A. Horses
   B. Crows
   C. A and B

5. Factors influencing the spread of Aedes mosquito-borne viruses globally include:

   A. Warming temperatures
   B. Urbanization
   C. Increasing global trade
   D. Increasing global travel
   E. All of the above

6. When taking a patient history, what does the "E" stand for in "Social-E"?

PETS AS SENTINELS

1. Animals may serve as sentinels for human environmental hazards due to:

   A. Greater exposure than humans
   B. Greater susceptibility
   C. Shorter latency
   D. All of the above

2. Rocky Mountain Spotted Fever is only found in a small area in the American West. TRUE or FALSE?

3. Professional communication between human health care providers and veterinarians:

   A. is routine in most communities
   B. is required by public health regulations

C. requires initiative on the part of human health care providers
D. is prohibited in some states

4. Sentinel cases of influenza A have been reported in:

   A. Cats
   B. Dogs

5. Reason(s) for a human health care provider to communicate with a veterinarian:

   A. Animal could infect your patient
   B. Animal could be allergy risk
   C. Some patients more likely to seek care for a pet than for themselves
   D. Potential for medication errors and misuse between human and veterinary meds
   E. All of the above

6. When taking a patient history, what does the "E" stand for in "Social-E"?

EMERGING ZOONOSES

1. Which of the following viruses is not a bat-borne pathogen?

   A. Rabies virus
   B. Rift Valley fever virus
   C. Marburg virus
   D. Ebola virus
   E. Nipah virus

2. Which of the following is TRUE about Nipah virus?

   A. It can be transmitted to humans from animals such as bats or pigs
   B. It can be transmitted from human-to-human
   C. There are no licensed treatments or vaccines available
   D. All of the above

3. Which of the following zoonotic viruses is known to spill over into humans most often?

   A. Nipah virus
   B. Ebola virus
   C. Lassa virus
   D. Marburg virus
   E. SARS-CoV

4. Which of the following zoonoses can be transmitted via animal bites and/or scratches?

   A. Bartonella
   B. Pasteurella
   C. Rat bite fever
   D. Capnocytophaga
   E. All of the above

5. For a patient with fever and altered mental status in South Asia, which of the following pathogens is least likely?

   A. Nipah virus
   B. Dengue virus

C. Yellow fever virus
D. Japanese encephalitis virus

6. When taking a patient history, what does the “E” stand for in “Social-E”?

<u>ECOANXIETY</u>

1. When taking a patient history, what does the “E” stand for in “Social-E”?

2. Symptoms of environmental anxiety are distinct from those in other anxiety disorders. TRUE or FALSE?

3. Ways to build host resilience to environmental anxiety include:
   A. Relaxation training
   B. Healthy lifestyle- exercise/sleep/diet
   C. Collective action about environmental change
   D. All of the above

4. Ways to manage patient environments to prevent worsening ecoanxiety include:
   A. Encourage Increased exposure to the negative aspects of environmental change in order to build resilience.
   B. Counsel to limit exposure to troubling environments such as disaster areas
   C. Counsel to limit exposure to news about environmental problems
   D. A and C
   E. B and C

5. Baseline diagnostic studies for a patient presenting with environmental anxiety:
   A. TSH (thyroid stimulating hormone)
   B. CBC (complete blood count), metabolic profile
   C. EKG (electrocardiogram)
   D. All of the above
   E. A and B

6. The Social-E history can include questions about the effect of the environment on patient’s mental health. TRUE or FALSE?

<u>WATER-RELATED DISASTERS</u>

1. List 4 potential direct health impacts of a disaster.

2. A 36 year old female presents to your emergency hospital in a remote disaster zone with extreme pain in her left thigh after she scraped her leg a week or more ago while going to get food for her family in poor, extremely muddy conditions. The leg has evidence of the small prior wound injury on the lateral aspect of the thigh but then has notable extensive surrounding erythema, edema of the extremity, and hemorrhagic bullae. No other noted injuries on exam. Patient is febrile, hypotensive to 80/60 and tachycardic to 120. Assume you have access to and capacity for all possible answer choices listed. What is your next step in management?

   A. X-ray of the left thigh, hip, knee
   B. Stabilize with antibiotics and resuscitation only
   C. Resuscitate and coordinate transfer out of country expected to take 12 hours
   D. Proceed to OR for immediate debridement

3. A 44 year old woman with a history of poorly controlled T2DM presents to a field clinic 4 days after an earthquake. She has a diffuse erythema over her medial left thigh with severe tenderness and crepitus on exam. Blood cultures are drawn and she is prepared to take to the OR for debridement. What antibiotic regimen is most appropriate for this patient?

   A. Doxycycline and ceftriaxone
   B. Piperacillin/tazobactam and vancomycin
   C. Clindamycin
   D. Amphotericin B

4. You are a member of an NGO team partnered with a local government's response to extreme flooding in India. While treating penetrating injuries in the impact phase, you are anticipating future needs. Which of the following is <u>not</u> your priority?

   A. Working with your team and government workers to obtain additional health care personnel
   B. Preparing for future cases of drowning
   C. Facilitating the monitoring and evaluation of O2 supplies
   D. Obtaining emergency food supplements

5. When taking a patient history, what does the "E" stand for in "Social-E"?

<u>FOOD SECURITY</u>

1. List <u>3 ways</u> in which climate change and other environmental factors can impact <u>food security</u>.

2. Name <u>1 tool</u> that can be used to screen for food insecurity.

3. By definition, patients who are <u>food insecure</u> will be <u>underweight</u> on physical exam. True or False?

4. List <u>3 signs</u> of malnutrition that can be detected on physical exam.

5. List <u>3 characteristics</u> of a Planetary Health Diet.

6. When taking a patient history, what does the "E" stand for in "Social-E"?

<u>REFUGEE HEALTH</u>

1. The term "forcibly displaced people" includes:
   A. Refugees
   B. Internally Displaced People
   C. Asylum seekers
   D. A and B
   E. All of the above

2. List 1 resource where you can find guidelines for the medical examination of newly arriving refugees.

3. List 3 screening tests that are recommended as part of the medical evaluation of newly arriving refugees.

4. Symptoms of Latent TB Infection (LTBI) include:
   A. Fever
   B. Cough

C. Night sweats
D. A, B, and C
E. None of the above, patients with LTBI are asymptomatic.

5. Climate change can contribute to many key risk factors for poor TB outcomes. List 1 of these factors.

6. When taking a patient history, what does the "E" stand for in "Social-E"?

HEAT-RELATED ILLNESS

1. List 1 pre-existing condition that would place a patient at higher risk for heat illness.

2. List 1 environmental risk factor for heat illness.

3. List 1 preventive measure that you would discuss with patients at high risk of heat illness.

4. List 1 clinical sign that differentiates heat exhaustion from heat stroke.

5. How would you manage a patient with heat stroke? List 1 thing that you would do to manage a patient with heat stroke.

6. When taking a patient history, what does the "E" stand for in "Social-E"?

II. TOPIC GUIDE FOR FOCUS GROUP DISCUSSIONS

Begin focus group discussion by asking for general impressions of the case.

If needed, potential follow-up questions include:

- What stood out to you about the case? What were the main learning points?
- What do you think about integrating this type of content into medical education?
  - Do you think it is important to learn about the relationships between environmental change and human health as part of your medical education? Why or why not?
  - What concerns would you have?
  - In what ways does this content overlap with your current curriculum?
- How could this content be best integrated into medical education? At what stage? In what format?
- What did you think about the case content? Is it useful for you clinical practice? If so, how? If not, why not?
- How could the case be improved?
- What did you think about the format of the case? The "Patient Case" section? The "Beyond the Clinic" section? The "Call to Action" section? Were each of these sections useful? How so? If not, why not? How could they be improved?
- How could the case delivery and/or format be optimized for residents?
- How could the overall curriculum be improved? What topics should be included?
